# Technology User's Training Is a Waif

**DOI:** 10.5812/ircmj.10305

**Published:** 2013-11-05

**Authors:** Marzieh Adel Mehraban, Marzieh Hasanpour, Ahmadreza Yazdannik, Sima Ajami

**Affiliations:** 1 Nursing and Midwifery Faculty, Isfahan University of Medical Sciences, Isfahan, IR Iran; 2 Medical Management & Information Sciences Faculty, Isfahan University of Medical Sciences, Isfahan, IR Iran

**Keywords:** Heath Care, Nursing Research, Qualitative Research, Nursing Education

## Abstract

**Background:**

In recent decades, health care systems has greatly influenced by the technology development. The technology is helpful to enhance quality of care, reduce costs and improve patient care if appropriate training be applied for technology users. Technology user’s training has been studied in some quantitative studies; however, a few investigators have studied the challenges which nurses’ experienced in this regard.

**Objectives:**

This qualitative study conducted to explain how nurses explain and perceive challenges in technology user’s training.

**Materials and Methods:**

This qualitative study was conducted in 2012 by using the content analysis technique in which data were collected through interview. Twenty-four nurses who were occupied in hospitals affiliated to Isfahan University of Medical Sciences were selected by using purposive sampling and in-deep semi-structured interviews (focus-groups and individual interviews) were done. The content of data was analyzed by the Zhang and Wildmouth’s method.

**Results:**

Initial concepts evolved after content analyzing and five main categories were emerged. Theses categories were “Everything and everyone teach” technology users but there are “No effective training”, “Uninformed resources”, and managers always “Rely on trial and error” and there are some “Learning Barriers”.

**Conclusions:**

The result of this study outlined important concerns of nurses regarding biomedical technology user training, and determined the need for further education to modernized healthcare system for promoting and expanding patient care.

## 1. Background

Like other systems in the society, the health system is growing very fast and technology appliance is getting inevitable more and more. A crossing through many hospitals show many technological devises if are used effectively, safely, and correctly can significantly improve patient care status (1). This issue leads to more attention to the healthcare technology in the beginning of the recent century.

Nursing practice has been altered considerably informatics and health care technology application (2). Technology ranges from simple using of a digital thermometer to complex electronically equipment and hospital information systems. Informatics and health care technology have revolutionized health care delivery and created a level of complexity never experienced previously by health care providers (3).

Introduction of technology into the health care means that there is no specialist nurse who does not use at least one form of health care technology in her/his profession daily. Therefore, it is important for nurses to improve their knowledge and skills about technology, which has the deep affects on technology application and its capabilities (4-7).

The more technology application in health care means that nurses and other health care providers need more information about technology, and there is a believe that better technology understanding will promotes it’s using in patient care as well as the quality, safety and costs of care (8).

At the core of applying new technology, the proper introduction of technology will improve its implementation and consequently patient care (9) and the Technology User Training is considered as one of the most important components for workforce empowerment and effective technology application. However, this is especially true but the nursing community is not sufficiently trained about technology application. Professional nurses leave university without adequate understanding many biomedical technologies and this leads to providing insufficient health care.

Studies showed that technology user training before and during its application is very important in nursing practice. The research on determinants influencing the success of the introduction of new technologies showed that only half of nursing staff perceived the introduction process as good or even very good (10).

According to Douglas “In nursing, we are still learning the world of technology and developing the strength of our voice in getting what we need and want from it. We are still evolving our understanding of technology, its nature as a tool, and its relationship to a bigger picture of outcomes, goals, and cultural and business objectives” (11), and successful technology implementation in system depends on good leadership. Therefore, we decided to conduct a research about technology user training in nursing, because we believe that using our study’s result may lead to formulate some solutions for technology problems.

Although there are many researches about technology achievement and technology user training, but we believe that these researches are insufficient because they were often carried out in developed countries like United State of America, United Kingdom, Japan and Germany. Few researchers, especially in nursing, studied technology user training in developing countries such as Iran. Consequently, conducting a research about technology user training seems essential and this research report focuses on it.

## 2. Objectives

This qualitative study conducted to explain how nurses explain and perceive challenges in technology user’s training.

## 3. Materials and Methods

This qualitative study was conducted in Isfahan, Iran, by using the content analysis. After getting the approval from the Ethics Committee of Isfahan University of Medical Sciences [No: 390214] In this research study, the criteria required that all participants be practicing registered nurses with a minimum of six months of nursing practice experience, who currently provide direct care to patients and families in the hospitals affiliated to the Isfahan University of Medical Sciences. Study population included nurses, head nurses, supervisors, and chief nursing managers who were selected using purposive sampling (maximum variation sampling). The researcher’s assumption was that these particular participants are representative sample of nurses of all ages and experiences and who would be able to articulate their understandings about technology user training. Exclusion criteria included having less than two years of experience, being discontent, a history of mental illness, lack of employment at the time of research, and applications for study leaving. The sampling was continued until the data saturation, E.g., when the researcher could not achieve a new code (12). In-depth semi-structured interviews [12 individual interviews and two focus-group methods in four and seven-person groups] were conducted using brainstorming and interview techniques with 23 participants. Interviewer was researcher who was familiar to interview and communication principles.

The interviews were done at calm and private places without disruption, and recorded by digital voice recorder during meetings. The participants' informed consent was received before the interview. To ease discussion, the interviewer used open-ended questions based on the previous nurses’ experience about technology user training if they had not spontaneously mentioned such elements. First, unstructured interview was started by a general question: “As a nurse, how did you train about technology application? And “what is your problem in use of technology related to training” Moreover, they were asked to express and explain their experiences about technology user training challenges. We considered providing feedback, making trust, and avoiding induce comments to the interviewee during the interview. Researchers used systematic and purposeful sampling to discover how nurses were trained for technology application until data had been saturated. In addition, we regarded heterogeneous sampling for collecting different views about technology user training. 

Interviews duration was about 30 - 90 minutes according to the participant's preference and information.Then researchers transcribed recorded interviews verbatim and consisted of totally 120 written pages in Microsoft Office Word.

Data analysis in this study consisted of seven steps as mentioned by the Zhang and Wildmouth’s method which involves a set of systematic and transparent procedures for processing data to support valid and reliable inferences. Some of the steps overlap with the traditional quantitative content analysis procedures, while others are unique to this method (13).

Step 1- Data preparation: audio files were transcribed using lyric maker capability in the jet audio software to reflect participants’ thoughts and behaviors. Then texts were transported into word processor for commenting and read and re-read several times by all researchers.

Step 2- Defining the unit of analysis: in this stage, we determined and highlighted each sentence, word, phrase or paragraph as coding unit by reviewing the text.

Step 3- Developing categories and a coding scheme: The deductive coding was done. Concepts were put into similar categories and then main categories were formed.

Step 4- Testing coding scheme on a sample of text: Researchers conducted coding individually. Then coding consistency was checked through an assessment of the inter-coder agreement during meetings. Revising of the coding rules continued until sufficient coding consistency was achieved.

Step 5- Coding of the whole text: We re-read the transcriptions again and again, and by reading the transcribed text segments back and forth we coded the themes and the sub -themes. Then all codes were transferred into Microsoft office OneNote, the conceptual codes were cleared, and the concepts categories were classified. Finally re-encoding was done. Every overlapped or unusual code was deleted.

Step 6- Assessing the coding consistency: In several stages, researchers verified and investigated the encoding process. When there was no agreement among researchers, re-encoding was performed until coding consistency was achieved which can also contribute to credibility. In addition, four PhD students reviewed and confirmed emerged codes. Researchers tried to use purposeful sampling and choosing participants with various experiences for increasing shedding light on research questions from a variety of aspects.

Researchers did not clearly insist on the findings’ transferability. However, procedures were explained clearly and obviously to help following up of the research process so that other researchers can judge the appropriateness of the research process. Recording and transcription of the participants’ interview, as well as coding and classifying of concepts by some expert nurses and academic members of nursing faculty who did not participate in the early stage of the research and were familiar with qualitative research, were other critical tactics for achieving credibility during the research process.

Step 7- Drawing conclusions from the coded data: Reading and re-reading data helped to discover characteristics and dimensions of categories and the relationship among themes. Results of these stages are provided in the results section.

A variety of tactics, as defined in the Research Ethics Application, were applied to the research process to ensure that all necessary ethical considerations were considered and incorporated into the research design. Ethics approval was obtained from the Joint Committee of the Isfahan Medical Science University Research and Ethics review board. Ethical considerations were also carried out with respect to the gift (Intravenous Therapy book which was written by researchers) given to all participants who agreed with participate in this study. Confidentiality of the prospective participants, researchers did also assign a research pseudonym to each of the participants, Ensure all consent forms, hand-written and electronically generated via e-mail were password protected and secured in a private and locked location, and Interviews were conducted in a mutually agreed on location that ensured privacy protection to the participant.

## 4. Results

This study aimed to investigate how is technology user training in nursing. Participants were 23 in total, 20 female, three male, participated from a variety of patient care settings. Ages of the participants ranged from 25 - 55 years (mean age = 38 years). Fifteen of the 23 participants were registered nurses who held direct care responsibilities to patients, three participants were head nurse, two of them were clinical nurse educator, and three participants were chief nurse manager. Each of the participants had nursing practice experience ranging from two to 28 years (mean = 13.5 years). All participants were educated in university in different level (refer to Table 1). 

**Table 1.** Participant Sample Demographics

Characteristics	No.
**Participants’ age, y**	
25 - 35	8
35 - 45	10
45 - 55	2
**Gender**	
Male	3
Female	20
**Clinical position**	
Nurse	15
Head nurse	3
Supervisor	2
Chief nurse manager	3
**Education**	
Bachelor	13
Master of nursing	2
M.Sc. student	8
**Years of nursing practice experience, y**	
Level	2- 28
Mean	13.5
**Clinical practice domain**	
Med/surg unit	5
Critical care unit	4
Internal unit	6
Nurse managers office	5
Emergency unit	3

Based on the experience of the participants, there are some challenges in technology user training process. We reached to about 80 interpretive meanings after data analysis, which we produced 50 common interpretive meanings after combination. These meanings were classified into 23 codes and five main categories were emerged in further data analysis. Table 2 shows these data. 

**Table 2.** Challenges in Technology User Training

Challenges in Technology User Training	
**Everything and Everyone Teach**	Chief nursing managers as trainer
	Supervisors as trainer
	Head nurses as trainer
	Colleagues as trainer
	Medical engineers as trainer
	Manufacturers as trainer
**No Effective Training**	Training not for all nurses
	Training not for all shifts
	No special time for training
	Training by ignorant people
	Training for acquiring rank
**Uninformed Resources**	Unaware engineers
	Unaware manufacturers
	No access to user guide
**Rely on Trial and Error**	Learning through experience
	Other institute technology user training' experiences as a learning model
	No feedback for training
**Learning Barriers**	Refusing by old nurses
	preoccupied nurses
	No master in English language
	No master in using computer
	Fear of failure

### 4.1. Everything and Everyone Teach

Participants mentioned that technology user training was informal and there was no identified responsible person or team for it. Sometimes, other nurses trained one another and in other cases other health care providers such as medical engineers trained them, or even they were trained just by reading catalogues and brochures. Often, chief nursing managers scheduled and followed up classroom training. In many cases, supervisors, especially educational supervisors were responsible for technology user training. They scheduled, coordinated, and followed up hours of training classroom and supervised it. Head nurses trained some other nurses about technology application too.

Plus many nurses stated that their colleague's guidance trained them about technology, especially nurses who work in evening and night shifts (when nurse managers were not available). 

One nurse stated about technology user training in her unit, as follows:

"For example, when a new dressing [he/she defined technology as new dressing] is introduced, company send a marketer to explain for head nurses about how this dressing should be used and then our head nurse explains it to us"(P7)

One participant, who was educational supervisor, explained about technology user training in hospital:

"In some cases, head nurse asks nurses who are in touch, to participate in the training classroom. They receive one or two- hour training class and then they start to use it" (P4)

Sometimes nurses told: 

"We learn about technology by reading catalogues" (P22, P13, P14)

"Everybody teach me how to use technology, there is no limitation, but sometimes, they do it wrong because they leant it by themselves not by knowledge"(P19)

This means although technology user training can support best performance, but the identified corresponding for it, is absent in hospitals and not always nurses have access to a specific resource to address their questions about using technology.

### 4.2. No Effective Training

More than half of nurses interviewed by investigators corroborated that there is no sufficient time for technology user training. Nurses complained that they had no opportunity to participate in all training classes. Nursing shortage often were forced the head nurse to choose just some staffs for training.

For example, one head nurse mentioned nursing shortage and inadequate training:

"There is nursing shortage. All of my staffs cannot participate in training classes because somebody should deliver nursing care so I had to send only some of them to learn and then they teach one another. Of course they cannot train each other’s as well as educators, but it is unavoidable" (P1)

Moreover, several participants stated that there was no proper time for education because nurses received the training either in day shift (while they are doing their duties) or in after night shift (when they are too tired to learn). Therefore, nurses are either under stress due to patient care or fatigued or distracted for effective learning.

A head nurse remarked about the training time:

"It is very good to have the proper training and time, but sometimes we have no sufficient time for training. We may apply new technology in our unit and just one or two nurses were trained for its using. It is ideal for us to have enough time for training enough users, and to make sure that everybody learns technology application.”(P10)

Five nurses described user training by a person who was unfamiliar with heath care environment and so education was not effective and applicable. They told:

"They do not know enough about accurate working with devices" (P17)

Therefore, the result showed that there is no effective and efficient time for technology user training.

### 4.3. Uninformed Resources

Many participants stated dissatisfaction with the system ability to meet their specific needs, answer their questions, and debug their faults during technology application. More than half of nurses stated that some medical engineers have no sufficient information about how technology should be used and cannot meet nurses’ training needs. They mentioned that sometimes a device might be out of use because nobody knows how to solve an existing simple deficit. Four participants explained that sometimes nurses use a new technology but there is no access to instructions for this product. Sometimes, instructions are in foreign language and users cannot use them easily.

One specialist nurse who worked in Intensive Care Unit (ICU) stated that:

“I remember when a monitoring device was introduced to us, we asked about some buttons and keys, and the engineers told us that it is not important for you to know” (P2)

### 4.4. Rely on Trial and Error

Six of the 23 interviewed nurses expressed dissatisfaction with the user training because they had no enough training and information about technology and had to use technology evens when difficulties arise during application. Three of participants noted that sometimes they accidentally discovered some capability of devices.

When we asked how technology users are trained in nursing, one nurse answered as: “For example during a night shift the monitor had some errors, I asked my colleagues about fixing it, but nobody were there to help me, and I had to use it as we presumed” (P6)

Some nursing managers expressed that they learned technology application and its accurate performance through visiting other health care services. They (especially nurse managers) mentioned that other hospitals are models for acquiring skills and knowledge about technology performance.

About seven participants explained that there was no assessment and feedback to measure user training effectiveness and to support nurses’ performance. One participant stated:

“Nobody knows how training is and does training program have sufficient effect on nurse's performances while it may provide very useful information for further education” (P12)

User (nurses) training, obviously, neither was designed well nor was evaluated based on measurable performance criteria. Nurses’ statements inferred that they did not receive effective training to interact safely and confidently with devices.

### 4.5. Learning Barriers

Another significant category was learning barriers. Some nurses’ statements inferred that they had no motivation for learning about technology and its application. Sometimes, older nurses were not motivated for technology user training. They thought that they have limited cognitive and physical capability to operate a complex device.

For example, a head nurse told:

“We announce a time for user training class. Young nurses give a warm welcomed but older nurses do not tend to participate and tell us that every things seem ok without using technology, it (technology) is not for us we are very old to use technology [and so on]” (P1)

Some nurses also complained about spending all their time with the technology instead of patients. They preferred not to know technology because then their managers would force them to use it. They believed technology pulls them further and further away from patient.

Inadequate knowledge about English language leads to lack of motivation for nurses too. They found medical devices, especially computers hard to apply. Therefore, they were not motivated to use computers and other devices, which needed English language knowledge.

In addition, fear of failure decreased nurses’ motivation for applying technology. Long time, which is required for fixing devices, cost, and interrupted patient care, was all reasons for nurses to avoid technology and its learning.

Head nurse of a surgery ward stated:

“Before I am being a head nurse, I was so… I mean I never liked learning new technology, I feared about applying computer, because I thought it will required repair and I didnot like learning about it, I resisted to new devices. But now I understand they are very useful for providing health care, they are very good” (P20)

## 5. Discussion

This study aimed to investigate the nurses’ experience and perceptions regarding the technology user training challenges in nursing practice. Results showed that technology user training in nursing is not adequate and effective in Iran. There is no systematic and regular process for nurse’s technology user training and it is unclear who is responsible for nurses training about technology. It is frustrating that nurses in this study recognized lack of their skills and support for using new technology whereas actual training of end user is a great opportunity to reinforce goals and address improvement (11). Technology is a multifaceted phenomena that extensively influences nursing history, contemporary nursing practice, and its future. Nurses are expected to practice in clinical environments where both the society and health care professions respected technical performance highly, more than ever before (14).

Technology has advanced so quickly in the last decades, but nurse's satisfaction is insufficient and patient care has not been improved. This is obvious that technology implementation is not effective by itself and required clear effective policy. Sometimes applying technology to an already broken structure can aggravate existing problems (11). This study showed that although there are many persons who participate in technology user training in nursing but there was not a well-known in charge or a team who analyse technology training needs and organize necessary training for nurses. In addition, hospitals had not special team for technology user training and just some staffs in hospitals did training activities besides to other duties that they have to perform.

Nurses always complained that they have lack of access to the informed resources, the organization had not tried to collect data about technology training needs, and there is no real supervision for technology training for nurses. It is essential for all organizations to have a strategic team, who meets nurses repeatedly during technology implementation, and discusses barriers or concerns. Nursing leaders should provide users with sufficient and proper training on the new technology to ensure its successful implementation. Managers should not assume that nurses have (or do not have) the necessary skills to apply the technology in practice because it does not mean that young staffs have more technology-related ability or conversely old nurses have the instinct to learn and apply the technology.

We found no study related to technology training in Iran to compare its results with results of the present study but in other country we found researches which confirm our findings. For example veer and her colleagues in their research found the same results in the Netherlands. Their study’s aim was to gain a better understanding of causes influencing the success of the introduction of new technologies as perceived by nursing staff. In that study results showed nurses most frequently mentioned they perceived support from colleagues in use of the new technology. Nurses also referred training and coaching is the most important factor associated with the introduction of a new technology. They reported that sometimes several weeks or months elapsed in training and the actual time in introducing technology (10).

Therefore, we should assess the training needs of each employee who will interact with the technology. A study in 2007 showed that nurses in Australia, Canada, UK, Singapore, and South Africa, often are concerned about technology user training too. They believed that time, and quality of training always were insufficient, and 61% of them preferred more education about technology in use (6). Furthermore, results of other studies showed there is more need about technology user training and it should be improved (5, 15, 16).

In our study, over half of participants believed that the system did not fully prepare them to work with new technologies and the training could have gone better. Nurses were inhibited from learning and using technology because of individual circumstances or personal deficiencies like unfamiliarity with English language, high workload, lack of computer-related knowledge, and fear of failure in using technology. Prior studies also have showed that nurses are poorly prepared to use biomedical technology and have identified numerous barriers that affect user training (16-20).

Taking a deeper dive about the nurses training into the sea of technology can provides significant insights about technology implementation. Although training and coaching is the most frequently mentioned factor associated with the successful introduction of a technological innovation (21), this study demonstrated that technology training is “waif” in clinical environment and there is no adequate attention to its different aspects. Any weakness in technology training can lead to waste funds and even patient injuries. Therefore, nursing leaders must promote technology management competencies and provide some programs to support training and development of the necessary skilled nurses.

### 5.1. Limitations

The limitations of this study are related to its design. In qualitative studies, quality would be affected by individual researchers’ skills, personal biases and idiosyncrasies and this may be affected our study too. However, all researchers had experiences in qualitative study. Thus, there is the need for further qualitative.
